# Camel milk consumption, perceptions, and knowledge of health and food safety among adults in Saudi Arabia: a cross-sectional study

**DOI:** 10.3389/fpubh.2026.1835528

**Published:** 2026-08-13

**Authors:** Jozaa Zaidan ALTamimi, Tahany Saleh Aldayel, Lujain A. Almousa, Dalal Aljabryn, Nawal Albadr, Shaista Arzoo, Reham I. Alagal, Nora Abdullah AlFaris

**Affiliations:** 1 Department of Sports Health, College of Sports Sciences & Physical Activity, Princess Nourah bint Abdulrahman University, Riyadh, Saudi Arabia; 2 Department of Health Sciences, College of Health and Rehabilitation Sciences, Princess Nourah bint Abdulrahman University, Riyadh, Saudi Arabia; 3 Department of Food Sciences and Nutrition, College of Food and Agriculture Sciences, King Saud University, Riyadh, Saudi Arabia

**Keywords:** camel milk, consumer perception, dairy consumption, food safety, nutrition knowledge, raw milk, Saudi Arabia, sustainable food systems

## Abstract

**Objective:**

Camel milk is a culturally important food in many arid regions and has gained increasing attention due to its distinctive nutritional composition and potential health-related properties. However, limited information is available regarding consumer perceptions, knowledge, and consumption patterns of camel milk in Saudi Arabia. This study aimed to assess camel milk consumption patterns, consumer knowledge and perceptions of pasteurized and unpasteurized camel milk, and factors influencing consumption among adults in Saudi Arabia.

**Methods:**

A cross-sectional survey was conducted between January and December 2025 using an online questionnaire distributed nationwide. A total of 2,176 adults aged 18 years and older participated in the study. Descriptive statistics were used to summarize consumption patterns and perceptions, while independent t-tests and one-way ANOVA were used to examine differences in knowledge scores across socio-demographic variables and a multivariable logistic regression model was used to identify factors associated with camel milk consumption.

**Results:**

Overall, 24.3% of participants reported consuming camel milk. Fresh camel milk was the most commonly consumed form, whereas processed camel milk products such as cheese, yogurt, and ice cream were consumed less frequently. Health-related beliefs, including perceived medicinal properties and high nutritional value, were among the strongest motivations for consumption. Taste preference, religious beliefs, and traditional practices also influenced consumption behavior. In contrast, sensory attributes, particularly taste and smell, were the main barriers reported by non-consumers. A substantial proportion of participants expressed positive perceptions of unpasteurized camel milk, associating it with naturalness and health benefits, despite known food safety concerns. Knowledge scores differed significantly according to age, education, income, region, and health status, but not by sex, marital status, or camel milk consumption status. Informal sources such as friends, relatives, and social media were the most common sources of information regarding camel milk.

**Conclusion:**

These findings highlight the need for targeted nutrition and food safety education addressing misconceptions about raw camel milk, alongside strategies to improve product availability, sensory quality, and consumer awareness to support safe and sustainable camel milk consumption in Saudi Arabia.

## 1 Introduction

Camels (*Camelus dromedaries*) represent an important livestock species in arid and semi-arid environments due to their remarkable ability to adapt to harsh climatic conditions, including high temperatures, limited water availability, and poor-quality feed resources. In these ecosystems, camels play a vital role in supporting food security and livelihoods by providing milk, meat, and transportation. Beyond their traditional role in pastoral systems, camel-related industries have gained increasing economic and cultural importance across the Arabian Peninsula. Recent Saudi national report highlighted the growing economic value of camel-based products and the expanding role of camel dairy production within regional food systems (1). Camel milk has long been consumed in many regions of the Middle East and North Africa and has recently attracted increasing scientific and commercial interest. The transition from traditional subsistence production toward more organized commercial dairy systems has contributed to growing consumer demand and expanding market availability of camel milk products in the Gulf region (2). Scientific interest in camel milk has also increased due to its distinctive nutritional composition and potential biological properties.

Camel milk also contains a wide range of nutrients and bioactive compounds that may contribute to its functional and nutritional properties (3). Recent studies have further highlighted its micronutrient composition and functional characteristics, which may influence its potential health benefits and industrial applications (4, 5). Camel milk is considered as highly nutritious milk due to the presence of health beneficial substances such as bioactive peptides, lactoferrin, immunoglobulins, lysozyme, monounsaturated and polyunsaturated fatty acids (3, 6). It possess many beneficial nutritional and therapeutic characteristics, and hypo-cholesterolemic, hypoglycemic, hypoallergic, immunostimulatory, antimicrobial, and anticarcinogenic properties (6–9). A systematic review suggests that the high levels of anti-inflammatory proteins found in camel milk may provide benefits to the stomach and help alleviate various gastrointestinal disorders (10). It also supports the regulation of various essential metabolic processes including digestion, absorption, and elimination (11).

Early investigations into the health-related effects of camel milk were primarily based on *in vitro* studies and limited animal and human trials (12). In addition, several studies have reported that the camel milk exhibits various bioactivities; however, much of this evidence is derived from experimental or preliminary clinical studies and requires further confirmation (6, 13, 14). Some studies have suggested that camel milk may serve as a potential alternative for individuals with cow’s milk allergy or lactose intolerance, although evidence remains limited and further clinical research is needed (15, 16).

Despite the increasing scientific interest in camel milk, consumer acceptance and consumption patterns may be influenced by multiple factors, including sensory characteristics, cultural practices, perceived health benefits, and economic considerations. Previous research has demonstrated that consumer knowledge, perceptions, and attitudes toward dairy products play an important role in shaping consumption behavior and market demand (17).

In Saudi Arabia, camel milk is consumed both through commercial retail markets and direct purchase from farms, where raw milk remains widely available. Camel milk contains valuable nutrients and has several potential health benefits; however, it may pose public health risks when consumed raw or unpasteurized primarily because of contamination with pathogenic microorganisms that can cause foodborne diseases (18). For example, contamination of camel milk with *Brucella abortus* has been reported in several regions (19). Behavioral studies have also shown that some consumers continue to consume raw milk despite awareness of potential risks, often due to traditional practices or perceived health benefits (20, 21). Although the nutritional properties and potential health effects of camel milk have been widely studied, limited research has examined consumer knowledge, perceptions, and consumption patterns in Saudi Arabia, particularly with regard to pasteurized and unpasteurized camel milk. Understanding these factors is important for informing public health strategies and food safety interventions. Therefore, the present study aimed to assess camel milk consumption patterns, consumer knowledge and attitudes, perceived benefits and risks, and factors influencing camel milk consumption among adults in Saudi Arabia.

## 2 Methods

### 2.1 Study design and participants

An online cross-sectional survey was conducted between January and December 2025 using a questionnaire distributed via online platforms. An online survey was used due to its cost-effectiveness, rapid data collection, and broad reach. Individuals aged 18 years and older were eligible to participate. Participants were recruited using an online survey distributed via social media platforms and email, including WhatsApp™, Twitter/X™, Facebook™, and LinkedIn™. Due to the online distribution strategy, a convenience sampling approach was used to reach participants across different regions of Saudi Arabia. A total of 2,176 participants completed the questionnaire and were included in the final analysis. Participation was voluntary and no financial incentives were provided. The first page of the survey included an information sheet outlining the study objectives and procedures, and electronic informed consent was obtained from all participants before they took part. No personally identifiable information was collected.

### 2.2 Sample size consideration

The minimum required sample size for cross-sectional studies is typically around 384 participants at a 95% confidence level and 5% margin of error, as suggested in population-based nutrition research (22). The sample size (2176) was considered adequate to ensure sufficient statistical power for population-level estimates and subgroup analyses.

### 2.3 Ethical considerations

The study was conducted in accordance with the ethical principles outlined in the Declaration of Helsinki. Ethical approval was obtained from the institutional research ethics committee (Number 24-0848). Electronic informed consent was obtained from all participants before survey completion.

### 2.4 Survey questionnaire

A self-administered, structured questionnaire was developed using Google Forms and was made available in Arabic. The questionnaire was designed based on extensive review of the literature related to camel milk consumption, perceptions, and health benefits (12, 23). The questionnaire comprised 25 items assessing participants’ sociodemographic characteristics, camel milk consumption practices, perceptions (attitudes) towards camel milk, and knowledge regarding camel milk. The practices section assessed actual consumption behaviors and habits, the attitudes section included statements reflecting beliefs and perceptions toward camel milk, while the knowledge section included objective questions assessing participants’ awareness of the health and safety aspects of camel milk (e.g., risks associated with consuming unpasteurized milk).

The questionnaire was pilot-tested among 30 adults to assess clarity and relevance, and minor modifications were made accordingly. Internal consistency reliability was evaluated using Cronbach’s alpha coefficient based on the main study data, which yielded a value of 0.83, indicating a good internal consistency among the questionnaire items and supporting its suitability for application in the study.

#### 2.4.1 Socio-demographic information

Participants provided socio-demographic data including age, sex, and marital status, educational level, and monthly income, region of residence, family size, and self-reported health status or presence of chronic diseases.

#### 2.4.2 Camel milk consumption

Participants were asked whether they had ever consumed camel milk and to report their current consumption status. Questions assessed frequency and duration of camel milk consumption, preferred types of camel milk products (e.g., full-fat milk, skimmed milk, yogurt, laban, cheese, cream, ice cream, and oggt), and preferred consumption methods (raw, boiled, pasteurized, or powdered). Participants were also asked about sources of camel milk and camel milk products and the use of camel milk in beverages and food preparation.

#### 2.4.3 Motivations for camel milk consumption

Motivations for consuming camel milk were assessed using a series of statements related to perceived nutritional value, medicinal properties, fat quality, sugar and sodium content, taste, religious beliefs, traditional practices, availability, and affordability. Responses were recorded using a Likert-type scale (always, often, sometimes, rarely, never), consistent with previous studies assessing consumer attitudes toward dairy products (17).

#### 2.4.4 Perceptions of unpasteurized and pasteurized milk

Participants’ perceptions of unpasteurized and pasteurized camel milk were evaluated using statements related to freshness, nutritional value, immune and digestive benefits, safety, shelf life, and risk of foodborne illness. Responses were recorded using an agreement-based Likert scale, as previously applied in studies assessing raw milk perceptions and safety awareness (23).

#### 2.4.5 Factors limiting camel milk consumption and proposed measures

Participants who reported not consuming camel milk were asked to identify barriers to consumption, including taste, smell, availability, price, spoilage, and safety concerns. Participants were also asked to suggest potential measures that could increase camel milk consumption, such as improving sensory quality, increasing availability and production, reducing price, and incorporating camel milk into popular food products, consistent with consumer behavior research (24).

#### 2.4.6 Health and nutritional benefits knowledge assessment

Knowledge regarding the health and nutritional benefits of camel milk was assessed using a structured set of questions with predefined correct answers. Correct responses were summed to generate a composite knowledge score for each participant, with higher scores indicating greater knowledge. This approach has been widely used in previous nutrition knowledge assessments (25). Knowledge regarding the health and nutritional benefits of camel milk was assessed using a structured set of questions with predefined correct answers. Knowledge items were scored using a scale ranging from 0 to 2, where 0 indicated an incorrect response, 1 indicated uncertainty, and 2 indicated a correct response. This scoring system was used to capture varying levels of understanding among participants, enabling a more comprehensive assessment rather than a binary classification.

#### 2.4.7 Sources of information on camel milk benefits

Participants were asked to identify their primary sources of information regarding the health and nutritional benefits of camel milk and its products. Response options included friends and relatives, social media, television, scientific evidence, healthcare professionals, and lack of interest in seeking information, in line with earlier consumer knowledge studies (17).

### 2.5 Statistical analysis

Data were analyzed using the Statistical Package for the Social Sciences (SPSS), version 25.0. Continuous variables were expressed as means ± standard deviations (SD), while categorical variables were presented as frequencies and percentages. Independent-sample *t*-tests and one-way analysis of variance (ANOVA) were used to examine differences in knowledge scores across socio-demographic and consumption-related variables and a multivariable logistic regression model was used to identify factors associated with camel milk consumption. Statistical significance was set at *p* < 0.05.

## 3 Results

### 3.1 Socio-demographic characteristics of the participants

A total of 2,176 participants completed the survey. The mean age of participants was 28.4 ± 11.2 years. The socio-demographic characteristics of the study population are summarized in Table 1. Females represented the majority of respondents. Most participants were from the Central administrative region, while smaller proportions were from other regions of the country. The largest age group was young adults aged 19–30 years, followed by those aged 31–50 years, whereas older age groups accounted for only a small proportion of the sample. Most participants were single, with approximately one-third being married.

**Table 1:** Socio-demographic characteristics of participants (*n* = 2,176).

Variables	*n*	%
Gender		
Male	332	15.25
Female	1844	84.742
Administrative region		
Central	1,673	76.884
Northern	92	4.2279
Eastern	215	9.880
Western	105	4.825
Southern	91	4.181
Age in years		
19–30	1,482	68.1
31–50	539	24.8
51–70	149	6.8
More than 70	6	0.3
Marital status		
Single	1,361	62.5
Married	721	33.1
Divorced	94	4.3
Education level		
Less than high school	138	6.3
High school	719	33
University	1,186	54.5
Post-Graduate	133	6.1
Monthly income (SAR)		
Less than 5,000	1,279	58.8
5,001–10,000	491	22.6
10,001–20,000	272	12.5
Higher than 20,000	134	6.2
Family members		
2	233	10.7
3–5	850	39.1
6–8	859	39.5
Higher than 8	234	10.8
Health status		
Healthy	1874	86.1
Blood Pressure	47	2.2
Diabetes	39	1.8
Cholesterol	57	2.6
Heart diseases	7	0.3
Cancer	6	0.3
Digestive system problems	61	2.8
Several health problems	85	3.9
Camel milk consumption status		
Camel milk consumers	529	24.3
Non camel milk consumers	1,647	75.7

Regarding educational level, more than half of the participants held a university degree, and around one-third had completed high school. Only a small proportion reported either lower or postgraduate qualifications. In terms of income, the majority reported earning less than 5,000 Saudi Riyal per month, with progressively fewer participants in higher income categories. Family size was most commonly between three and eight members, whereas smaller or very large families were less frequent. Most participants reported being healthy, while only a small minority indicated the presence of chronic conditions such as hypertension, diabetes, high cholesterol, or other health problems. Overall, 24.3% of respondents (*n* = 529) were classified as camel milk consumers, whereas the majority (75.7%, *n* = 1,647) were identified as non-consumers.

### 3.2 Practices related to camel milk consumption

#### 3.2.1 Camel milk consumption patterns

**Figure 1:**
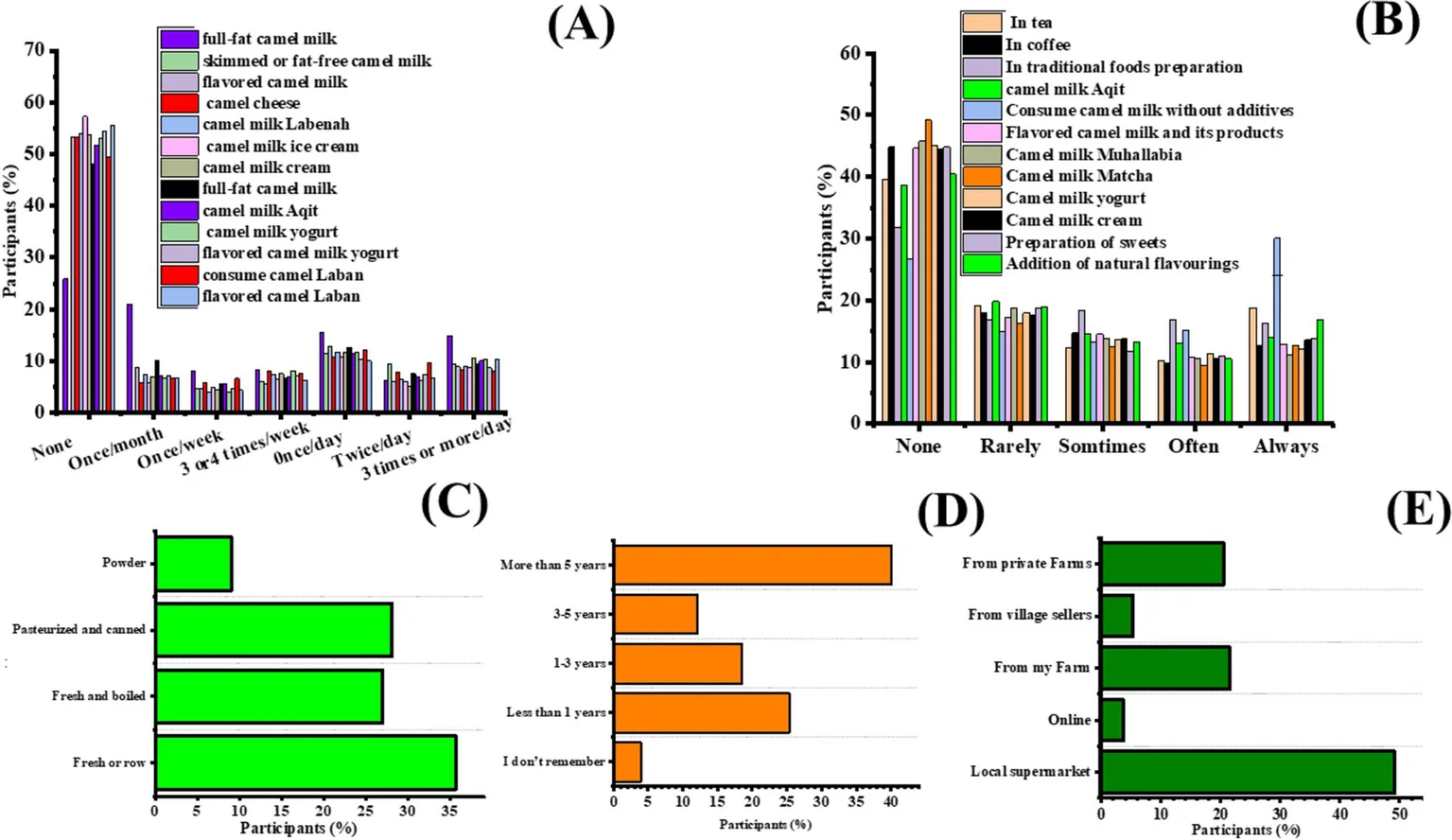
Camel milk consumption habits among participants who reported drinking camel milk (*n* = 529). **(A)** Camel milk/its product consumption habits. **(B)** Application of camel milk in functional food products. **(C)** Camel milk and product consumption patterns. **(D)** Duration of camel milk consumption. **(E)** Sources of camel milk and products.

A total of 529 participants reported consuming camel milk and provided detailed information about their consumption habits (Figures 1A–E). As shown in Figure 1A, nearly one-quarter of participants reported not consuming full-fat camel milk, while more than half indicated no consumption of flavored camel milk. For most processed camel milk products, including cheese, labneh, ice cream, cream, yogurt, flavored yogurt, laban, and flavored laban, the largest proportion of respondents selected “none.” However, a minority reported regular consumption (once per day or more), particularly for full-fat camel milk and certain fermented products such as laban and yogurt.

Regarding functional applications (Figure 1B), most participants reported that they never used camel milk in tea, coffee, or traditional food preparation. Consumption without additives showed comparatively higher “always” responses than flavored or mixed preparations, while the addition of natural flavorings was generally uncommon.

Figure 1C shows that fresh or raw camel milk was the most preferred form of consumption, followed by boiled or pasteurized milk, whereas powdered camel milk was the least preferred option. As illustrated in Figures 1D, a substantial proportion of participants had been consuming camel milk for more than 5 years, while shorter consumption durations were less frequent, and only a small minority could not recall when they started consuming camel milk. Figure 1E indicates that local supermarkets were the main source of camel milk and its products, followed by private farms, whereas online purchases and village sellers represented only minor sources.

#### 3.2.2 Factors associated with camel Milk consumption

Multivariable logistic regression identified gender and education as significant predictors of camel milk consumption (Table 2). Males were more likely to consume camel milk than females (OR = 2.115, *p* < 0.001), likely reflecting cultural or behavioral factors. Education was also positively associated with consumption (OR = 1.169, *p* = 0.048), suggesting that greater awareness may influence dietary choices. No significant associations were found for age, region, income, social status, family size, or health status (*p* > 0.05), indicating that consumption is driven more by knowledge and gender-related factors than by economic or demographic variables. These findings highlight the need for targeted awareness strategies to promote camel milk consumption, particularly among underrepresented groups.

**Table 2:** Multivariable logistic regression analysis of factors associated with camel milk consumption.

Variables	OR [Exp(B)]	*p*-value
Gender	2.115	<0.001
Administrative region	1.049	0.329
Age in years	0.929	0.529
Social status	0.97	0.816
Education status	1.169	0.048
Monthly income	0.888	0.069
Family members	0.887	0.066
Health status	1.021	0.528
Constant	0.67	0.483

### 3.3 Perception (attitude) towards camel milk

#### 3.3.1 Motivations for camel milk consumption among consumers

A high proportion of participants strongly agreed that camel milk has high nutritional value and medicinal properties (Table 3). More than one-third of respondents reported always preferring camel milk because of its perceived medicinal properties, while 31.4% reported always preferring it due to its high nutrient content Similarly, 27.6 and 27.2% of participants indicated that they always preferred camel milk because of its low sugar content and good fat quality, respectively.

**Table 3:** Consumers’ reasons for preferring camel milk over other types of milk (*n* = 529).

Nutritional value and Medicinal properties					
Response category	High nutrient content and low-fat content	Its fat content is good	It has a low sugar content	It has a low sodium content	Medicinal properties
Never (%)	22.117	25.5198	25.330	27.788	19.281
Rarely (%)	10.207	11.909	11.720	12.854	10.775
Sometimes (%)	16.068	18.525	17.391	16.2570	14.177
Often (%)	20.226	16.824	17.958	17.958	18.903
Always (%)	31.379	27.221	27.5992	25.1417	36.862

Other reasons					
Response category	Religious practice	Taste preference	Availability	Affordable price	Tradition
Never (%)	21.928	23.629	30.056	31.379	24.952
Rarely (%)	10.018	10.964	14.177	12.665	11.909
Sometimes (%)	15.879	14.933	15.689	18.147	16.257
Often (%)	16.446	14.177	20.037	17.202	17.391
Always (%)	35.727	36.2948	20.037	20.604	29.489

Preference based on low sodium content was slightly lower, with 25.1% reporting “always” and 18.0% reporting “often.” Religious beliefs were also a strong motivator, with 35.7% of respondents indicating that they always preferred camel milk for this reason. Taste preference was another important factor, with 36.3% reporting always preferring camel milk because of its taste. Traditional beliefs and practices also influenced consumption, with 29.5% reporting “always” and 17.4% reporting “often.”

In contrast, availability and affordability appeared to be less influential factors. Only 20.0 and 20.6% of participants, respectively, reported always preferring camel milk because of availability or affordable price, whereas higher proportions reported rarely or never selecting these reasons.

#### 3.3.2 Perceptions of unpasteurized and pasteurized camel milk

**Figure 2:**
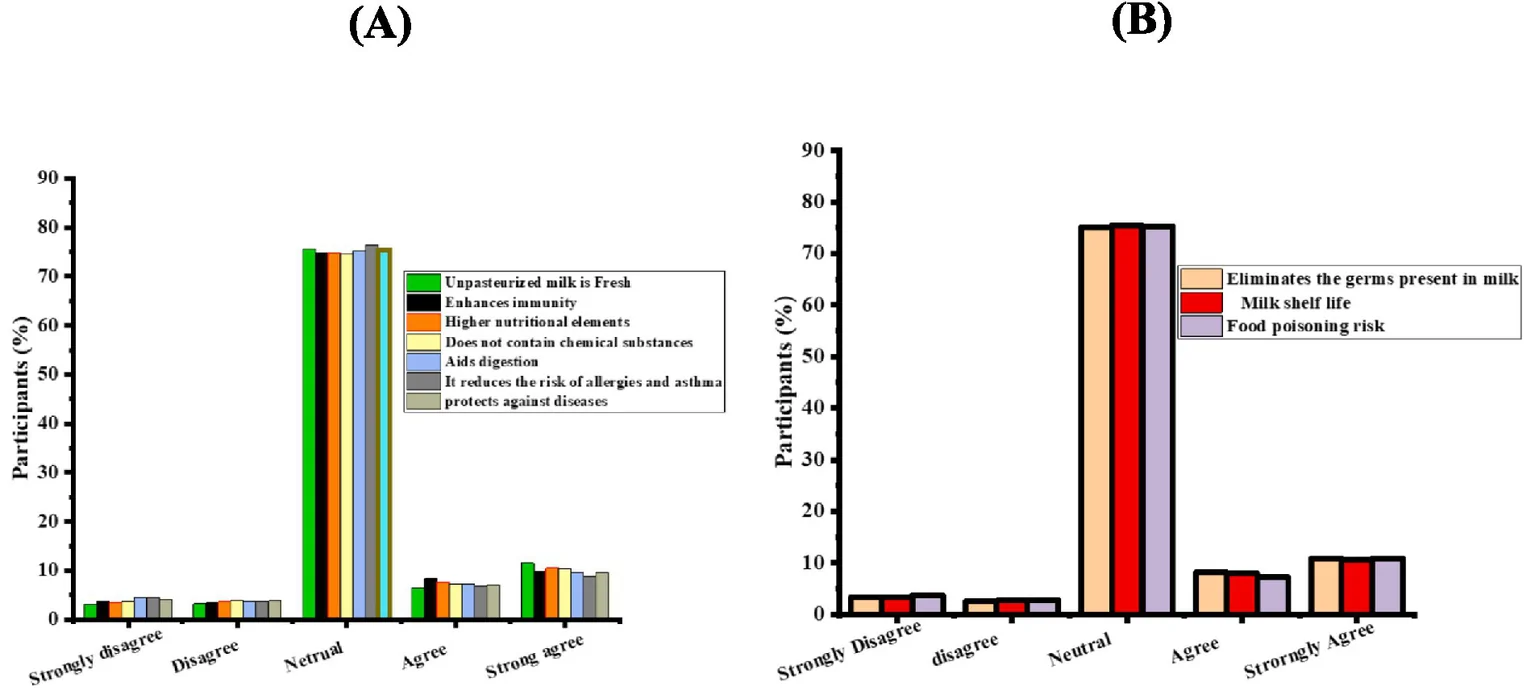
Participants’ perceptions of unpasteurized and pasteurized camel milk. **(A)** Unpasteurised milk. **(B)** Pasteurised milk.

Approximately half of the participants reported consuming unpasteurized camel milk either regularly or occasionally, while the remaining participants indicated that they did not consume it. As illustrated in Figure 2A, perceptions of unpasteurized camel milk were largely centered on its natural characteristics and perceived health benefits. Although many participants selected neutral responses, positive perceptions (agree and strongly agree combined) were more common than negative responses. Many respondents associated unpasteurized milk with freshness, absence of chemical additives, improved digestion, enhanced immunity, and higher nutritional value, reduced risk of allergies and asthma, and protection against certain diseases.

In contrast, perceptions of pasteurized camel milk were primarily related to safety and preservation (Figure 2B). Most participants selected neutral responses, while agreement responses were higher than disagreement responses. Participants commonly associated pasteurized milk with the elimination of harmful microorganisms, extended shelf life, and a reduced risk of foodborne illness. Overall, unpasteurized camel milk was more frequently perceived in terms of naturalness and potential health benefits, whereas pasteurized camel milk was mainly associated with safety and storage advantages.

#### 3.3.3 Factors limiting camel milk consumption and proposed strategies

As presented in Table 4, sensory characteristics were the main barriers among participants who did not consume camel milk or its products. Dislike of taste was the most frequently reported reason, either alone or in combination with other factors such as unpleasant smell and concerns about rapid spoilage. In contrast, dislike of smell alone was reported by only a small proportion of participants.

**Table 4:** Reasons for non-consumption of camel milk and participants’ suggestions to increase camel milk and its products consumption (*n* = 2,176).

Reasons for non-consumption of camel milk	*n*	%
Dislike of its taste	463	21.277
Dislike of its smell	101	4.641
High price	31	1.424
Not available in the supermarkets near my home	126	5.790
Not available in large enough quantities to find it anytime	64	2.941
Spoils quickly	25	1.1488
Not applicable to this question because I consume it	368	16.91
Dislike of its taste; Dislike of its smell	493	22.6562
Dislike of its taste; High price	30	1.3786
High price; Not available in the supermarkets near my home	12	0.551
Dislike of its taste; Dislike of its smell; Spoils quickly	458	21.047
Dislike of its taste; not available in sufficient quantities to be found at any time.	1	0.0459
High price; not available in the supermarkets near my home	1	0.0459
Not available in the supermarkets near my home; not available in sufficient quantities to be found at any time.	1	0.0459
Not available in the supermarkets near my home; not available in sufficient quantities to be found at any time; fast spoilage.	2	0.0919
Participants’ suggestions to increase camel milk and its products consumption		
Provide camel milk and its products in larger quantities	105	19.84877
Make camel milk and its products available in most markets and points of sale	46	8.695652
Improve the taste and smell of camel milk and its products	69	13.04348
Make its price reasonable and affordable	21	3.969754
Incorporate it into the production of popular foods and products	26	4.914934
Provide camel milk and its products in larger quantities; make them available in most markets and points of sale; make its price reasonable and affordable; incorporate it into the production of popular foods	169	31.94707
Provide camel milk and its products in larger quantities; improve the taste and smell of camel milk and its products; make its price reasonable and affordable	93	17.58034

Availability-related barriers were less frequently reported, and price was rarely identified as a sole reason for non-consumption. Overall, these findings indicate that sensory factors—particularly taste and smell—were the primary reasons for avoiding camel milk, whereas cost, availability, and shelf-life concerns played comparatively minor roles.

Participants also suggested several strategies that could increase camel milk consumption. The most frequently suggested single measure was increasing product availability in larger quantities, followed by improving taste and smell and expanding market distribution. Price reduction and incorporating camel milk into commonly consumed food products were less frequently reported as individual solutions.

Notably, a considerable proportion of respondents emphasized the need for combined improvements, including increased production, wider availability, affordable pricing, and product innovation. Overall, these results suggest that a comprehensive, multifaceted approach addressing sensory quality, accessibility, affordability, and product development may help promote greater consumption of camel milk and its products.

### 3.4 Knowledge of camel Milk

#### 3.4.1 Knowledge of health and nutritional benefits

The mean knowledge score regarding the health and nutritional benefits of camel milk varied across socio-demographic characteristics. No significant differences were observed between males (0.98 ± 0.12) and females (0.95 ± 0.04; *p* = 0.20), marital status (*p* = 0.118), or camel milk consumption status (*p* = 0.39).

However, knowledge scores differed significantly by administrative region (*p* < 0.001). Participants from the Southern (1.78 ± 0.30) and Western regions (1.06 ± 0.19) had higher scores than those from the Northern (0.47 ± 0.12) and Eastern regions (0.65 ± 0.11), which may reflect regional differences in camel milk accessibility and traditional consumption practices. Significant differences were also observed according to age (*p* = 0.002), education (*p* < 0.001), income (*p* < 0.001), and health-related conditions (*p* = 0.037). Younger participants, individuals with higher education and income levels, and those without chronic diseases demonstrated higher knowledge scores (Table 5).

**Table 5:** Knowledge score of health and nutritional benefits of camel milk among participants (*n* = 2,176).

Variables	Mean (Score)	Std. Error	*p*-value*
Gender			
Male	0.982	0.1164	0.200
Female	0.951	0.044	
Administrative region			
Central	0.970	0.047	
Northern	0.465	0.1178	
Eastern	0.653	0.107	0.0000
Western	1.064	0.191	
Southern	1.778	0.297	
Age (years)			
19–30	1.038	0.051	
31–50	0.875	0.082	0.002
51–70	0.471	0.1152	
More than 70	0	0	
Marital status			
Single	1.010	0.052	
Married	0.8365	0.070	0.118
Divorced	1.095	0.223	
Education level			
Less than high school	1.237	0.174	
High school	0.829	0.066	0.000
University	0.934	0.056	
Post graduate	1.549	0.213	
Monthly income			
Less than 5,000	0.946	0.054	
5,001–10,000	0.951	0.085	0.000
10,001–20,000	0.690	0.098	
Higher than 20,000	1.601	0.207	
Have health-related qualifications			
Healthy	1.003	0.045	
Blood Pressure	0.559	0.214	
Diabetes	0.128	0.089	
Higher Cholesterol	0.815	0.288	0.037
Heart diseases	0.714	0.461	
Cancer	1.05	0.502	
Digestive system problems	1.070	0.281	
Several health problems	0.532	0.1695	
Camel milk consumers			
Yes	0.894	0.088	0.389
No	0.977	0.0470	

*The *p*-values indicate the statistical significance of the independent t-test and one-way ANOVA test.

#### 3.4.2 Sources of information on camel milk benefits

Among participants who reported seeking information about camel milk, friends and relatives were the most frequently reported sources, followed by social media and television. Scientific evidence was cited by only 4.1% of participants, while healthcare professionals such as doctors or nutritionists were the least frequently reported sources.

**Figure 3:**
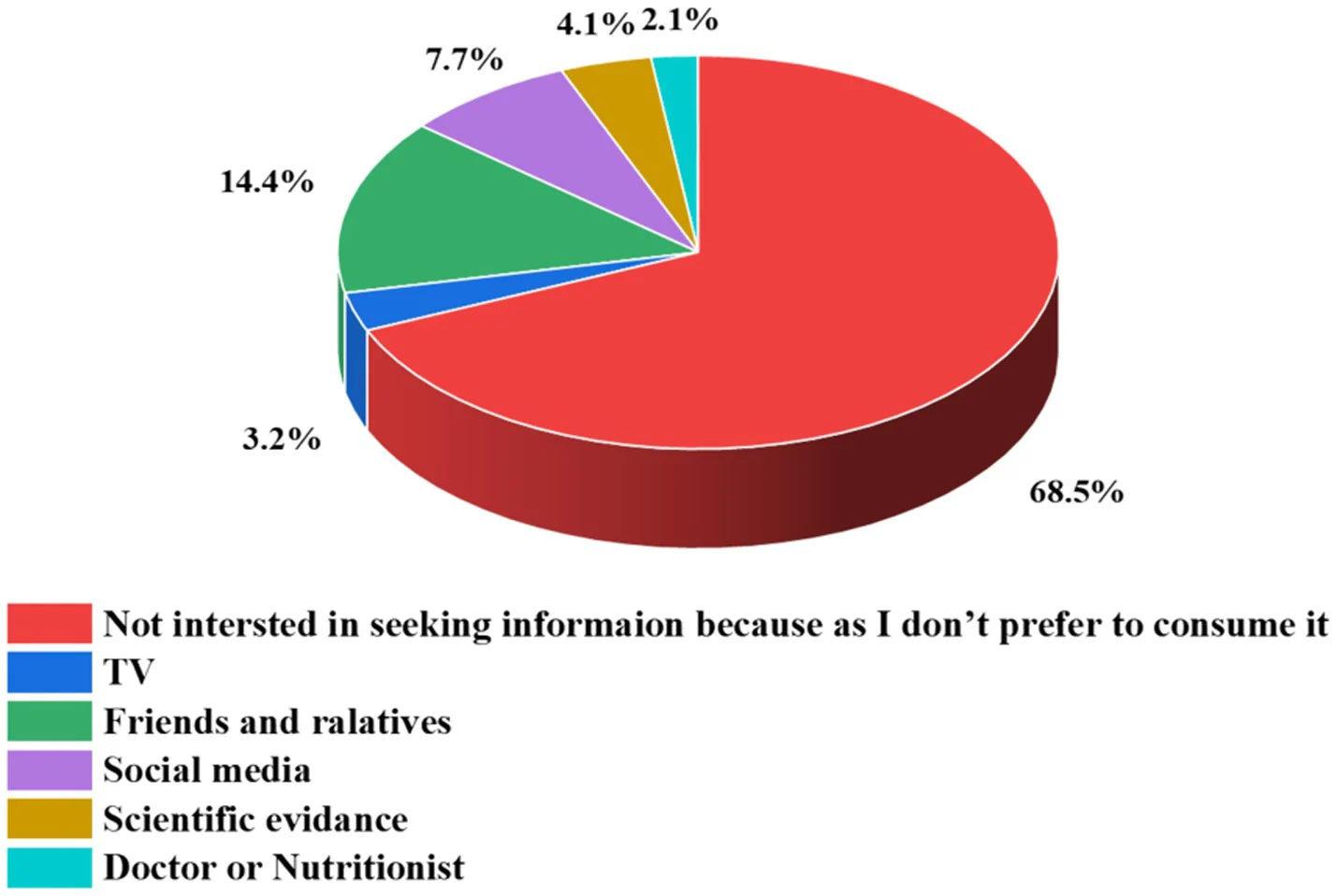
Sources of information on the health and nutritional benefits of camel milk and its products among participants.

In addition, many participants reported that they were not interested in seeking information about camel milk or its benefits because they did not prefer to consume it (Figure 3). Overall, these findings indicate that informal and non-scientific sources were more commonly relied upon than professional or evidence-based sources.

## 4 Discussion

Camel milk has long been consumed in many arid and Middle Eastern regions as an important traditional food and nutritional resource. More recently, scientific and public interest in camel milk has increased because of its distinctive composition, bioactive compounds, and potential health-related properties (4, 5). Despite this growing interest, evidence on consumer perceptions, motivations, and barriers to camel milk consumption in Saudi Arabia remains limited. The present study contributes to this area by examining camel milk consumption patterns, perceptions, and knowledge among adults in Saudi Arabia. Overall, the findings suggest that camel milk remains culturally valued and positively perceived, but regular consumption is still limited and appears to be shaped by sensory preferences, perceived health benefits, and regional and socio-demographic differences.

In the present study, fresh camel milk was consumed more frequently than processed camel milk products such as yogurt, cream, cheese, ice cream, and Aqit. This finding is consistent with previous reports indicating that camel milk is predominantly consumed in liquid form in camel-producing countries, whereas processed derivatives remain less common (26). The relatively low consumption of processed camel milk products may be due to limited market availability, and lack of consumer familiarity (27). Compared with bovine milk, camel milk has distinct physicochemical and techno-functional properties that may affect the manufacture and stability of dairy products, which may partly explain the narrower range of widely available commercial products (28, 29).

Although many participants had consumed camel milk, the proportion of current consumers was relatively modest. This pattern is broadly consistent with previous regional research showing that cultural familiarity does not necessarily translate into regular consumption (2, 17). One possible explanation is that favorable cultural and health perceptions may coexist with practical and sensory barriers that limit habitual intake. In the present study, participants commonly identified taste and smell as key determinants of consumption, suggesting that sensory acceptance remains central to consumer behaviors. This finding is important because it indicates that the expansion of camel milk consumption may depend not only on health messaging, but also on improving palatability and product format.

Regional differences were also observed in knowledge scores, with participants from the Southern and Western regions showing higher mean scores than those from the Northern and Eastern regions. These differences may reflect variation in local food culture, exposure to camel production systems, and familiarity with camel milk. However, these findings should be interpreted cautiously because the study sample was not evenly distributed across regions, and the Central region was more strongly represented than others. Because the survey was distributed online using convenience sampling, and the participation from the central region was higher than from other regions, this may limit the representativeness of regional comparison.

Perceived nutritional and medicinal value emerged as major motivations for camel milk consumption. Many participants believed that camel milk has high nutritional value and beneficial health properties, including favorable fat quality, low sugar content, and potential medicinal effects. These beliefs are consistent with the growing literature describing camel milk as a source of proteins, micronutrients (30–32) and bioactive components with possible antioxidant, antimicrobial, and immunomodulatory functions (3, 6, 13). However, although camel milk has shown promise in experimental and selected clinical contexts, the strength of evidence for many therapeutic claims remains variable and should be interpreted with caution (12). For this reason, public perceptions of camel milk as a medicinal food may currently be stronger than the level of evidence supporting some of these claims.

Religious and traditional beliefs also played an important role in shaping preferences for camel milk. This is not unexpected in the Saudi context, where camel milk carries long-standing cultural and symbolic value. Its milk is mentioned to in the Quran through the depiction of Paradise, where “rivers of milk the taste of which never changes” are promised, emphasizing milk as a symbol of pure, persistent, and profuse nourishment for all (33).

Camel milk is characterized by a salty taste, which may be ascribed to the various factors such as camel’s diet, lactation stage (34) and the decrease in key milk components and increase in chloride levels in milk collected from dehydrated camels (35). In this study, taste functioned as both a facilitator and a barrier: some participants strongly preferred camel milk because of its taste, whereas others avoided it for the same reason. This dual role highlights the heterogeneity of consumer experience and suggests that sensory optimization and product diversification may be important strategies for improving acceptability among non-consumers and occasional consumers.

An important finding of this study was the continued positive perception of unpasteurized camel milk. Many participants associated raw camel milk with freshness, naturalness, higher nutritional value which is in accordance with previous studies (2, 36). Another reason mentioned by the consumer was that it aids in digestion and similar response was reported earlier in a cross section study in UAE (2). A pilot study reported that as compared with pasteurized milk the raw milk did not lessen the lactose malabsorption or lactose intolerance symptoms among adults positive for lactose malabsorption, contradicting common claims that it improves digestion (37). It is predominantly valued for its better digestibility in the gastrointestinal system because of the small size of fat globules and its hypoallergenic properties (18). Such perceptions are likely influenced by cultural traditions and informal health beliefs. Similar attitudes toward the perceived benefits and risks of raw milk in general have been reported in previous studies (20, 21). However, these perceptions should be considered alongside established food safety concerns. Raw milk may act as a vehicle for foodborne pathogens and zoonotic infections, like *Brucella abortus* contamination (19) in raw camel milk. *Staphylococcus aureus*, *Escherichia coli* and *Salmonella*, in raw camel milk samples procured from street vendors and farm has also been reported (38). Moreover, Müller *et al*., (39) reported that *Mycobacterium bovis* infection can cause tuberculosis (TB) in humans, and the high TB rates in parts of East Africa with low HIV prevalence but high livestock exposure suggest that bovine TB from livestock or raw milk should be investigated (40, 41). The nutritional quality and microbiological contamination of raw camel milk from Jeddah farms and street vendors were assessed by Abdulsalam *et al*. (38). The study revealed pathogenic bacteria and increased bacterial contamination in vendors milk, highlighting potential public hazards associated with consuming raw camel milk. In addition, evidence suggests that pasteurization does not result in nutritionally meaningful losses that would justify the health risks associated with raw milk consumption (42, 43).

In a systematic review, it has been reported that, children, pregnant mothers, and other immune-surprised individuals were highly susceptible to infections associated with raw milk consumption (44). In the context of public health, unpasteurized camel milk may transmit zoonotic and foodborne pathogens, which may pose a risk of infection to consumers, especially if hygiene and storage conditions are inadequate (45). The persistence of favorable attitudes toward unpasteurized camel milk therefore indicates a need for culturally sensitive food safety education that addresses misconceptions while respecting traditional practices. Increasing consumer knowledge has been identified as an important strategy for reducing risks associated with raw milk consumption (46). Therefore, public health strategies should emphasize pasteurization, hygienic milk handling, regulatory oversight, and consumer awareness.

Another notable finding was the limited reliance on scientific or professional sources of information. Friends and relatives, social media, and television were more commonly cited than healthcare professionals or scientific evidence. This pattern may help explain why favorable beliefs about camel milk coexist with limited objective knowledge and with persistent beliefs in the superior benefits of raw milk. It also suggests that future educational efforts should not rely solely on formal healthcare settings, but should also engage with the communication channels that consumers already use. The present study also showed that knowledge scores differed significantly by age, education, income, region, and health status, but not by sex, marital status, or camel milk consumption status. Unlike these findings, a previous study (2) reported that knowledge scores differed significantly by gender and consumption status, indicating that localized socio-cultural and environmental factors, are the main drivers of awareness of camel milk. In this study, camel milk consumption was not associated with knowledge and this point is important because it suggests that consuming camel milk does not necessarily imply better factual knowledge about its health and nutritional properties. These findings support the need for targeted education strategies, particularly for groups with lower measured knowledge.

### 4.1 Limitations

This study has several strengths, including a large sample size and broad coverage of topics related to consumption patterns, perceptions, motivations, and knowledge. Nevertheless, some limitations should be acknowledged. First, the cross-sectional design does not allow causal relationships to be established. Second, compared to face-to-face method, it may introduce selection bias, limited inclusion of those without internet access, lower internet usage by older as compared to younger populations and lacks interviewer support for clarifying responses. Third, the data were self-reported and may therefore be subject to recall bias and social desirability bias. In addition, the regional distribution of participants was not uniform, which may limit the generalizability of regional comparisons. Despite these limitations, the large sample size provides valuable insights into camel milk consumption patterns and perceptions in Saudi Arabia. The sample was predominantly female, which may limit the generalizability of gender comparisons.

## 5 Conclusion

This study provides important insights into camel milk consumption patterns, perceptions, and knowledge among adults in Saudi Arabia. Although camel milk is widely perceived as nutritious and culturally significant, regular consumption remains limited among a large proportion of the population. Sensory characteristics, particularly taste and smell, were identified as the main barriers to consumption, while perceived health benefits and traditional beliefs were major motivations among consumers. The findings also highlight a continued preference for unpasteurized camel milk among some consumers despite known food safety risks. These results emphasize the need for targeted nutrition and food safety education, as well as strategies aimed at improving product accessibility, sensory quality, and consumer awareness. Further research should explore consumer acceptance of innovative camel milk products and evaluate the effectiveness of educational interventions aimed at promoting safe and sustainable camel milk consumption. From a sustainable food systems perspective, camel milk represents an important locally produced dairy resource in arid environments. Promoting safe and culturally acceptable camel milk consumption may contribute to strengthening regional food systems, supporting local livestock production, and improving dietary diversity in arid and semi-arid regions such as Saudi Arabia.

## Data Availability

The original contributions presented in the study are included in the article/supplementary material, further inquiries can be directed to the corresponding author.
